# High hydrostatic pressure induces pro-osteoarthritic changes in cartilage precursor cells: A transcriptome analysis

**DOI:** 10.1371/journal.pone.0183226

**Published:** 2017-08-16

**Authors:** Kevin Montagne, Yasuko Onuma, Yuzuru Ito, Yasuhiko Aiki, Katsuko S. Furukawa, Takashi Ushida

**Affiliations:** 1 Department of Mechanical Engineering, University of Tokyo, Tokyo, Japan; 2 Biotechnology Research Institute for Drug Discovery, National Institute of Advanced Industrial Science and Technology, Tsukuba, Japan; 3 Department of Bioengineering, University of Tokyo, Tokyo, Japan; University of Umeå, SWEDEN

## Abstract

Due to the high water content of cartilage, hydrostatic pressure is likely one of the main physical stimuli sensed by chondrocytes. Whereas, in the physiological range (0 to around 10 MPa), hydrostatic pressure exerts mostly pro-chondrogenic effects in chondrocyte models, excessive pressures have been reported to induce detrimental effects on cartilage, such as increased apoptosis and inflammation, and decreased cartilage marker expression. Though some genes modulated by high pressure have been identified, the effects of high pressure on the global gene expression pattern have still not been investigated. In this study, using microarray technology and real-time PCR validation, we analyzed the transcriptome of ATDC5 chondrocyte progenitors submitted to a continuous pressure of 25 MPa for up to 24 h. Several hundreds of genes were found to be modulated by pressure, including some not previously known to be mechano-sensitive. High pressure markedly increased the expression of stress-related genes, apoptosis-related genes and decreased that of cartilage matrix genes. Furthermore, a large set of genes involved in the progression of osteoarthritis were also induced by high pressure, suggesting that hydrostatic pressure could partly mimic *in vitro* some of the genetic alterations occurring in osteoarthritis.

## Introduction

During normal physical activity, physiological contact pressures in human articular cartilage vary between 0 and several MPa, reaching around 20 MPa during more intense activities [1][2]. Due to the high water content of cartilage (around 70%), most of the compressive load is sustained by the interstitial fluid [3], providing cartilage its high stiffness under compression and protecting the collagen-proteoglycan matrix from excessive deformation. As a result, it is assumed that one of the main physical stimuli sensed by chondrocytes embedded in the cartilage matrix is hydrostatic pressure (HP).

At the cellular level, moderate HP has been shown to promote cartilage differentiation by increasing the expression of cartilage matrix proteins such as aggrecan and type 2 collagen [4][5], and down-regulating the expression of matrix-degrading enzymes such as MMP-1 through a mechanism involving the transcriptional regulator CITED2 [6]. In cultured bovine chondrocytes, moderate pressures have no obvious effects on the cytoskeleton [7] and cyclical pressures even reverse the adverse effects of IL-1β in human chondrocytes [8]. *In vivo*, moderate joint loading through physical exercise has been shown to prevent the onset and slow the progression of osteoarthritis [9]. Conversely, excessive HP has been shown to induce opposing, detrimental effects on cartilage and chondrocytes, with increased apoptosis, decreased extracellular matrix synthesis, increased expression of stress- and inflammation-related genes as well as matrix-degrading enzymes [4][5][10]. High pressure also destabilizes the cytoskeleton [7] and induces changes in chondrocyte morphology reminiscent of those observed in osteoarthritic cartilage [11], illustrating the essential role of hydrostatic pressure mechanotransduction in the homeostasis of cartilage. In order to identify genes sensitive to high HP, Sironen et al. performed a cDNA array analysis of chondrocyte cell lines submitted to high HP (30 MPa) for up to 12 h [12]. However, this study focused on less than 600 genes and no global analysis of the effects of hydrostatic pressure on chondrocytes has been carried out so far.

In this study, we used whole genome microarrays to identify new genes modulated by high HP in the mouse ATDC5 chondrocyte precursor cell line. After 1, 4 and 24 h, pressurization under 25 MPa was found to modulate the expression of several hundreds of genes, including newly identified mechano-sensitive genes.

## Materials and methods

### Cell culture

Mouse ATDC5 chondrocyte progenitor cells were obtained from the Japanese Collection of Research Bioresources Cell Bank and cultured in DMEM/F12 (Life Technologies) supplemented with 5% of FBS (Nichirei) in a humidified incubator under a 5% CO_2_ atmosphere. Culture medium change was carried out every other day.

### Cell pressurization

**Fig 1:**
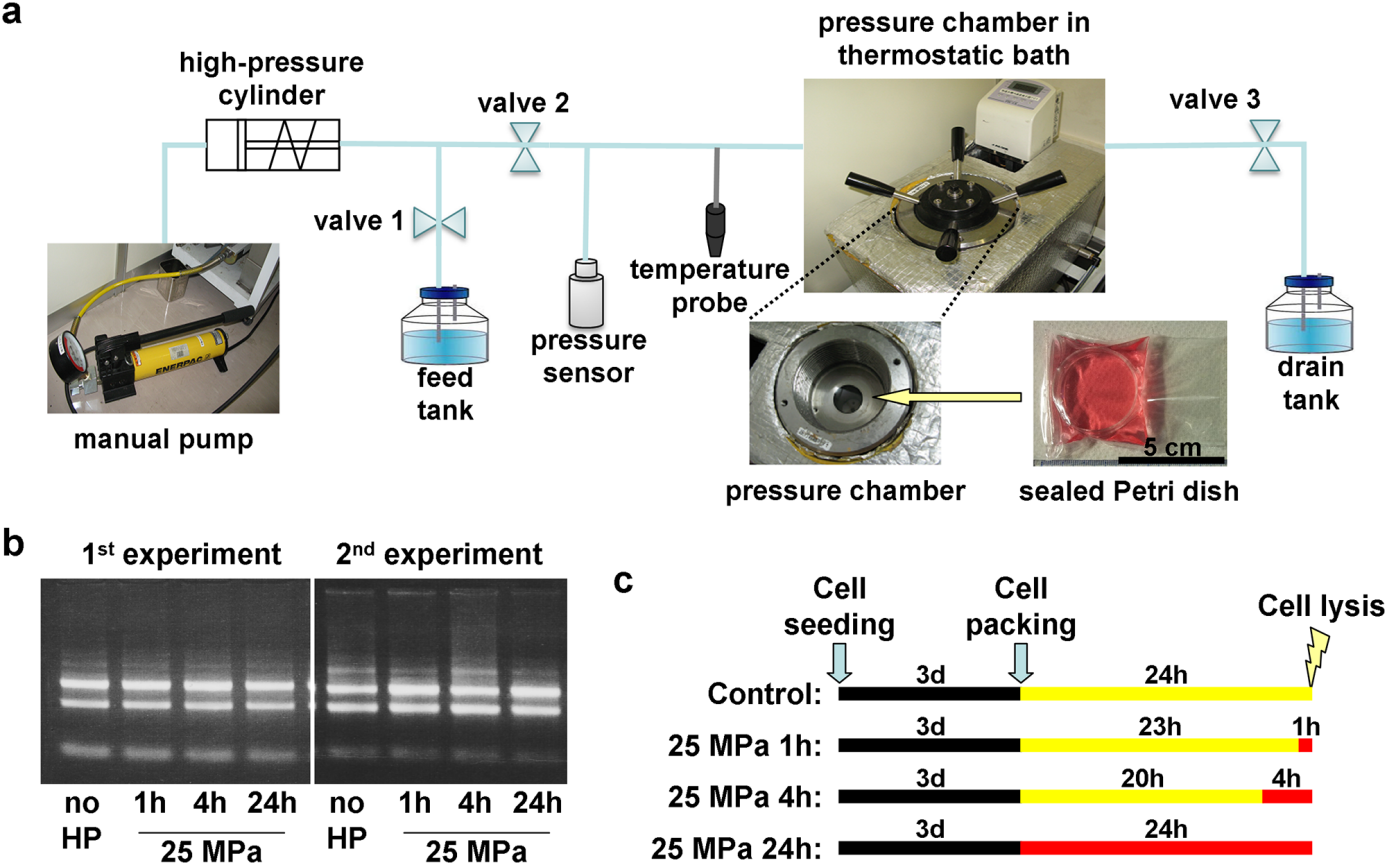
Experimetal setup. (a) schematic diagram of the pressurizing equipment. During pressurization, cells in Petri dishes sealed in polyethylene bags are placed in the pressure chamber, valve 3 is closed and water is pumped into the chamber through valve 2. (b) Ethidium bromide staining of RNA used for the microarray experiments separated in a 1% agarose gel. 500 ng of total RNA was run in each lane. (c) schematic diagram of the time course experiments: the yellow and red bars respectively represent the time the packed cells are placed in the water bath and in the pressure chamber under 25 MPa.

High hydrostatic pressure was applied for up to 24 h using a mechanical system (Fig 1a) consisting of a pressure chamber placed inside a water bath set at 37°C, and connected to a high-pressure cylinder operated by a manual pump. ATDC5 cells seeded at 2.10^4^ cells.cm^-2^ in 35 mm Petri dishes and cultured for 3 days until the cells formed a confluent monolayer were sealed in polyethylene bags (Seisan Nippon Sha Ltd) filled with 20 mL of culture medium and placed in the pressure chamber where a constant 25 MPa pressure was applied. The internal pressure and temperature of the chamber were continuously monitored by a pressure sensor. Unpressurized cells were sealed in similar bags and placed in a metallic column of the same dimensions as the pressure chamber in the same water bath. For each experiment, four dishes were used. In order to obtain the control, 1 h, 4 h and 24 h pressurized samples, at the start of the experiment, one dish was pressurized and the three remaining dishes were placed in the water bath; 20 hours later one dish was moved from the water bath to the pressure chamber; 3 hours later, one dish was moved from the water bath to the pressure chamber; then, 1 hour later, all samples were collected at the same time (Fig 1c).

### Cell count

Cells seeded on Petri dishes at 10^5^ cells.cm^-2^ were left to grow for three days and pressurized, or not, for the last 6 or 24 h. After fixation for 15 min with 4% paraformaldehyde, cell nuclei were stained with DAPI. Five pictures were taken randomly and the cells counted manually. Three independent experiments were carried out.

### Microarray experiments

The transcriptome of pressurized and unpressurized ATDC5 cells was analyzed using the SurePrint G3 Mouse Gene Expression v2 8x60K Microarray Kit (G4852B, Agilent) containing just over 56,000 probes (representing 27,122 genes and 4,578 long non-coding RNAs). Two time-course experiments (consisting of control, 1 h HP, 4 h HP and 24 h HP samples) were carried out, producing 8 RNA samples. The quality of the RNA was checked by agarose gel electrophoresis and found to be satisfactory, with two clear bands and no sign of degradation (Fig 1b). The RNA was then reverse-transcribed, labeled with Cy3 and hybridized to one microarray according to the manufacturer’s instructions. After hybridization, fluorescence signals were measured with a G2505C microarray scanner system (Agilent).

### Microarray data analysis

The raw data on each chip was normalized to the 75^th^ percentile of measurements taken from that chip. Genes, whose expression under pressure was more than doubled or halved compared to the control (fold change >2 or <0.5) in both experiments, were considered to be modulated. A rank product test was performed to assess the significance of the gene modulations found in the microarray. Venn diagrams were produced using Venny2.1.

Functional clustering of modulated genes was performed using DAVID 6.7 to identify over-represented groups of functionally-related genes.

Network analysis was first carried out using EGAN 1.5; genes significantly modulated with a p value<0.015 were selected to generate preliminary networks; subsequently, the genes identified by EGAN as being part of a network were then input into STRING 10.0 for confirmation. Interactions with a combined score above 0.9 were then used to generate reader-friendly networks with Cytoscape 3.4.0 [13].

Comparison between our data and previously published microarray data on surgically induced OA was carried out as follows: five microarray studies were selected on surgically induced OA, which included three mouse models (Gardiner et al. [14], Bateman et al. [15], Loeser et al. [16]) and two rat models (Wei et al. [17], Appleton et al. [18]), and genes modulated at least 2-fold were identified and compared to our list of modulated genes.

### Real-time PCR

Total RNA was extracted using Trizol (Invitrogen) and cDNA was synthesized from 500 ng of RNA using ReverTra Ace qPCR RT Master Mix with gDNA Remover (Toyobo). Real-time PCR was then carried out using Thunderbird SYBR qPCR Mix (Toyobo) in a StepOnePlus real-time PCR system (Life Technologies). *Rpl13a* was used as the reference gene. The primer sequences are listed in Table 1.

**Table 1:** Primers used for real-time PCR.

Gene	Forward primer	Reverse primer	Amplicon size (bp)
*Rpl13a*	TCTGGAGGAGAAACGGAAGGA	GGTTCACACCAAGAGTCCATTG	151
*Acan*	TTGGAGATCCAGAACCTTCG	TGTGCTCGATCAAAGTCCAG	164
*Adamts4*	GATCCAGCTAGGAGCTGTGC	TGCATGGCTTGGAGTTATCA	115
*Adamts5*	ACGGCATTATTGGCTCAAAG	GGGATCCTCACAACGTCAGT	130
*Arntl*	CTGCAGTGAATGCTTTTGGA	GCCACTGTAGTTGCTGGTCA	143
*Cd14*	AACCTGGAAGCCAGAGAACA	CAGAAGCAACAGCAACAAGC	134
*Cited2*	CATCGGCTGTCCCTCTATGT	TCTGCCATTTCCAGTCCTTC	106
*Col2a1*	CTCATCCAGGGCTCCAATGA	TCCTTCAGGGCAGTGTATGTGA	74
*Ctgf*	GAACTGTGTACGGAGCGTGA	TGGTATTTGCAGCTGCTTTG	173
*Ctsk*	CCAGTGGGAGCTATGGAAGA	CTCCAGGTTATGGGCAGAGA	118
*Cytl1*	GCTACTCTCGGATGCTGACC	AAGCCACGAAGTCTCTCAGC	159
*Ddit3*	CTGCCTTTCACCTTGGAGAC	GGACGCAGGGTCAAGAGTAG	162
*Errfi1*	CAATCTGAACTCCCCTGCTC	CCTTGGAGATGGACCACACT	106
*Fosl1*	TCTGGCCTATCCCCAGTACA	CCTTTCTTCGGTTTCTGCAC	182
*Gadd45a*	GAAGACCGAAAGGATGGACA	GCAGGCACAGTACCACGTTA	143
*Hbegf*	GACCCATGCCTCAGGAAATA	AGAGTCAGCCCATGACACCT	128
*Nfil3*	AAGGACCCATTGATGGATGA	GCTGCATCAGAAAGGACCTC	114
*Ptgs2*	GCTGTACAAGCAGTGGCAAA	TTCTGCAGCCATTTCCTTCT	144

### Statistics

All graphs show the mean +/- standard deviation. The statistical significance of the differences induced in gene expression by HP was assessed using a two-tailed unpaired t-test. A rank product test was carried out for microarray results.

## Results

### High HP strongly affects the gene expression profile of ATDC5 cells

**Fig 2:**
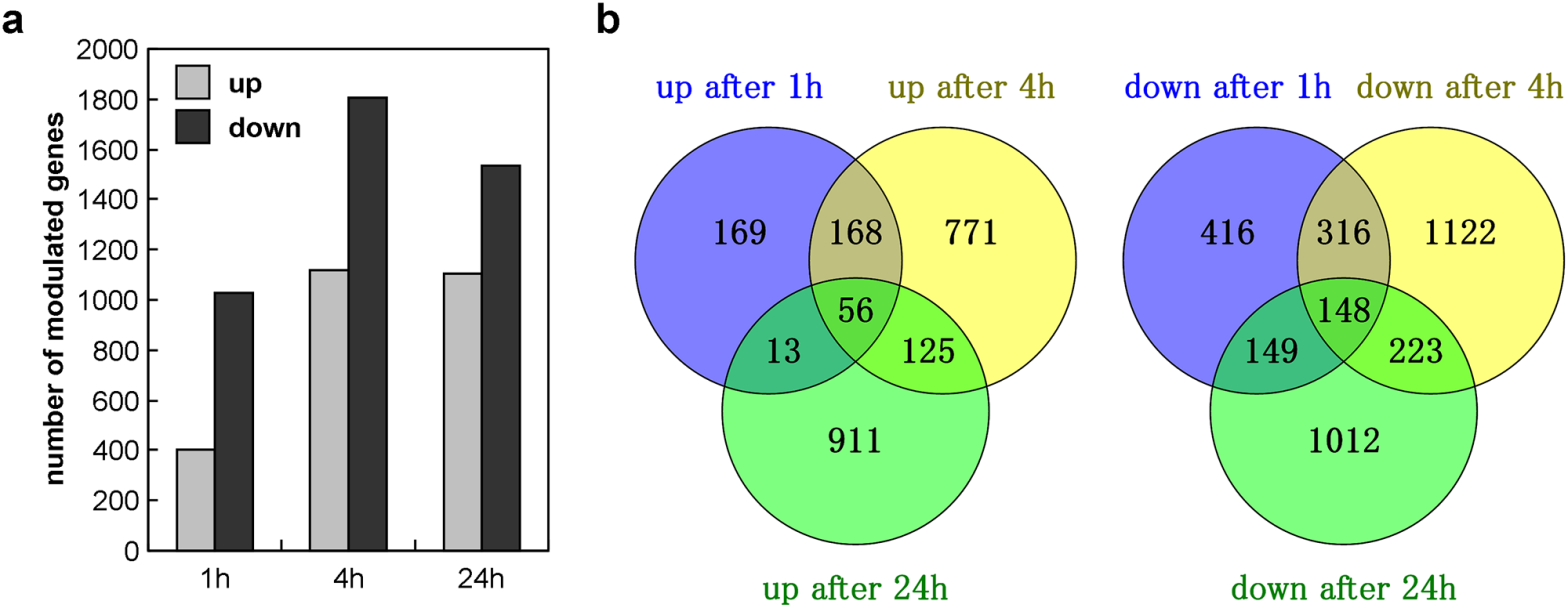
High HP modulates hundreds of genes. (a) number of genes up- and down-regulated at least 2-fold after 1, 4 and 24 h of pressurization. (b) Venn diagrams showing the overlap between genes up-regulated (left) and down-regulated (right) at different time points.

Within 1 h of pressurization, several hundred genes were already modulated by high HP. The number of modulated genes increased after 4h and remained stable after 24 h (Fig 2a).

**Fig 3:**
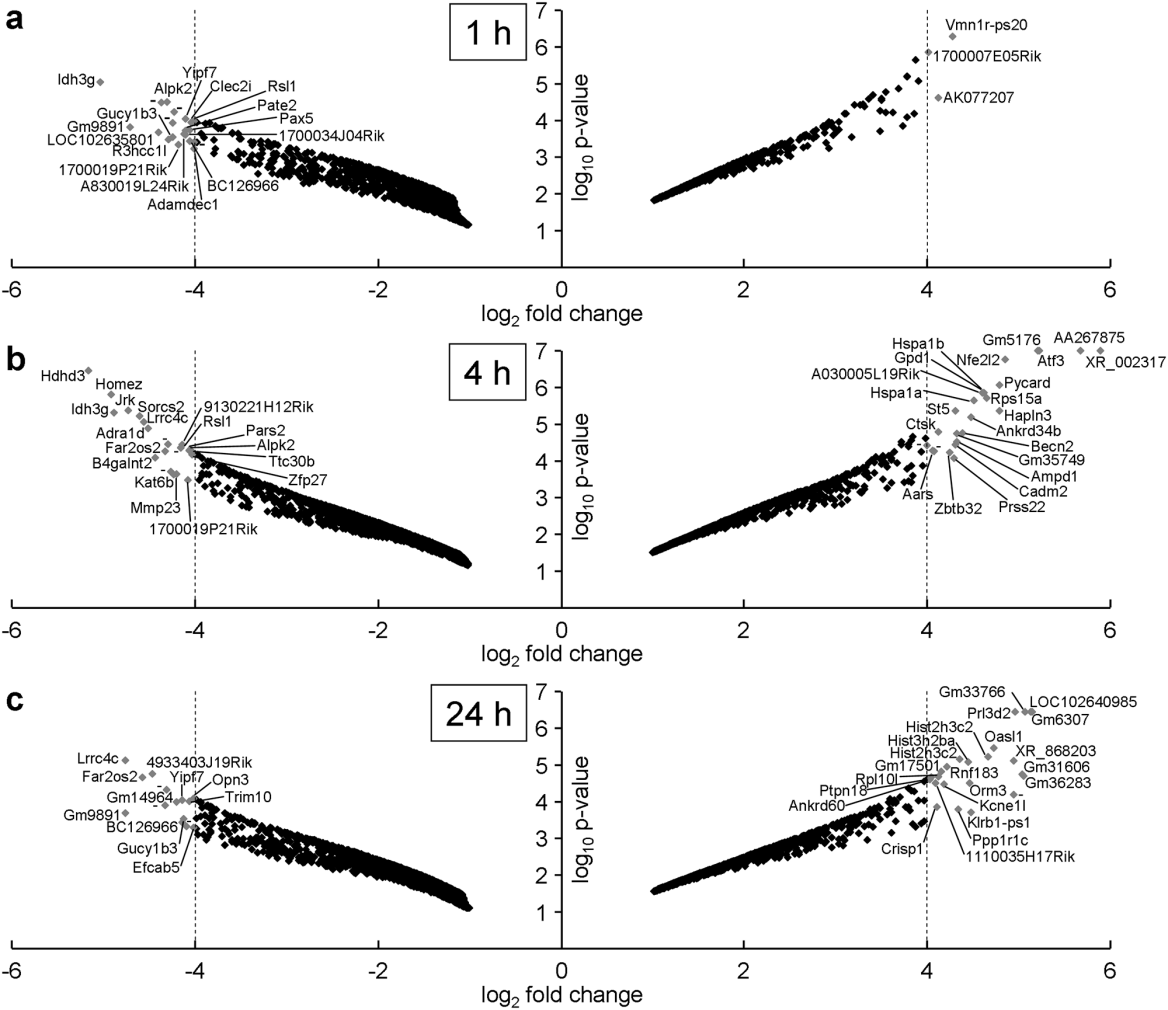
Volcano plot analysis of modulated genes at each time point. Volcano plots showing genes modulated with a fold change above 2 of below 0.5 after a) 1 h, b) 4 h or c) 24 h of pressurization. The base 2 logarithm of the fold change for each gene is plotted against the base 10 logarithm of the p-value obtained from the rank product analysis. The names of the genes with a fold change greater than 16 or less than 0.0625 (represented by the vertical dotted lines) are written next to the corresponding points.

Comparison of the modulated gene lists over time showed that about half the early modulated genes were also modulated after 4 h. The genes modulated after 24 h were quite different from those modulated at 4 h (less than 20% overlap), though 204 genes were modulated throughout from 1 h to 24 h of pressurization (Fig 2b). Nine genes down-regulated at least 16-fold (*1700019P21Rik*, *Alpk2*, *Far2os2*, *Gm9891*, *Gucy1b3*, *Idh3g*, *Lrrc4c*, *Rsl1*, and *Yipf7*) were found to be stably down-regulated over several time points (Fig 3). Table 2 shows the ten most significantly up- and down-regulated genes for each time point.

**Table 2:** Most significantly up- and down-regulated genes.

**Genes modulated after 1 h of pressurization**								
	**UP**				**DOWN**			
**#**	**Gene Symbol**	**Entrez Gene ID**	**mean fold change**	**p-value**	**Gene Symbol**	**Entrez Gene ID**	**mean fold change**	**p-value**
1	Vmn1r-ps20	666928	19.4	5.29E-07	Idh3g	15929	0.039	8.99E-06
2	1700007E05Rik	114672	16.2	1.41E-06	Alpk2	225638	0.054	3.17E-05
3	Hspa1b	15511	14.6	2.26E-06	Yipf7	75581	0.094	8.95E-05
4	Nfe2l2	18024	13.7	6.17E-06	Rsl1	380855	0.12	1.07E-04
5	Hspa1a	193740	13.7	6.52E-06	Clec2i	93675	0.12	1.15E-04
6	Rps15a	267019	15	8.28E-06	Gm35204	102638709	0.12	1.15E-04
7	Gm5176	382421	12.9	1.37E-05	4933403J19Rik	71021	0.073	1.18E-04
8	A030005L19Rik	77922	10.6	2.04E-05	Fut9	14348	0.096	1.21E-04
9	AK077207		17.5	2.43E-05	Vmn1r212	171275	0.086	1.39E-04
10	Vmn1r37	171183	11.3	2.48E-05	Gm9891	791316	0.21	1.52E-04
**Genes modulated after 4 h of pressurization**								
	**UP**				**DOWN**			
**#**	**Gene Symbol**	**Entrez Gene ID**	**mean fold change**	**p-value**	**Gene Symbol**	**Entrez Gene ID**	**mean fold change**	**p-value**
1	Gm5176	382421	36.8	1.00E-07	Hdhd3	72748	0.03	3.52E-07
2	Atf3	11910	37.4	1.00E-07	Homez	239099	0.042	1.59E-06
3	Nfe2l2	18024	28.8	1.76E-07	Jrk	16469	0.038	4.23E-06
4	Pycard	66824	27.7	8.81E-07	Idh3g	15929	0.043	4.93E-06
5	A030005L19Rik	77922	24.3	1.41E-06	Sorcs2	81840	0.041	5.99E-06
6	Gpd1	14555	24.4	1.41E-06	Lrrc4c	241568	0.043	8.64E-06
7	Hspa1b	15511	24.5	1.41E-06	Adra1d	11550	0.075	1.32E-05
8	Rps15a	267019	25.2	1.94E-06	9130221H12Rik	77124	0.058	3.60E-05
9	Hspa1a	193740	22.8	2.29E-06	Rsl1	380855	0.062	4.30E-05
10	Hapln3	67666	27.7	4.41E-06	Pars2	230577	0.06	5.06E-05
**Genes modulated after 24 h of pressurization**								
	**UP**				**DOWN**			
**#**	**Gene Symbol**	**Entrez Gene ID**	**mean fold change**	**p-value**	**Gene Symbol**	**Entrez Gene ID**	**mean fold change**	**p-value**
1	Gm6307	622283	35.5	3.52E-07	Lrrc4c	241568	0.038	7.40E-06
2	Prl3d2	215028	31.1	3.52E-07	4933403J19Rik	71021	0.046	1.74E-05
3	LOC102640985	102640985	35.1	3.52E-07	Far2os2	77841	0.098	2.22E-05
4	Gm33766	102636793	33.5	3.52E-07	Tas1r2	83770	0.23	2.66E-05
5	Oasl1	231655	26.4	3.34E-06	4732419C18Rik	100042484	0.17	2.68E-05
6	Hist2h3c2	97114	25.5	5.82E-06	Opn3	13603	0.068	8.05E-05
7	Hist2h3c2	97114	20.4	6.70E-06	Olfr947-ps1	257924	0.065	8.93E-05
8	Hist3h2ba	78303	21.8	8.11E-06	Yipf7	75581	0.1	9.04E-05
9	Rnf183	76072	18.5	1.09E-05	Trim10	19824	0.087	9.85E-05
10	Gm17501	100216343	17.7	1.48E-05	Gm14964	100008567	0.14	1.02E-04

### High HP adversely affects the cartilage phenotype

A search for over-represented functional groups with DAVID 6.7 indicated that high pressure induced cartilage de-differentiation and cell death in ATDC5 cells (Table 3): after 1 h of pressurization, genes involved in cellular response to stress and proteolysis were up-regulated, whereas genes classified as part of the primary cilium, a cell projection with a major role in chondrogenesis[19], were down-regulated; after 4 and 24 h, genes involved in cell death/growth arrest were induced, whereas genes involved in the extracellular matrix were down-regulated after 24 h of HP.

**Table 3:** Over-represented gene functional categories.

**genes UP-regulated after 1 h**			
**#**	**Gene Ontology Term**	**Category**	**Enrichment score**
1	nucleotide binding	Molecular function	3.81
2	response to organic substance	Biological process	3.31
3	chromosome organization	Biological process	2.96
4	proteolysis	Biological process	2.08
5	cellular response to stress	Biological process	1.55
**genes DOWN-regulated after 1 h**			
**#**	**Gene Ontology Term**	**Category**	**Enrichment score**
1	ion binding	Molecular function	9.61
2	monosaccharide metabolic process	Biological process	1.47
3	positive regulation of catalytic activity	Biological process	1.15
4	vesicle-mediated transport	Biological process	1.14
5	cilium	Cellular compartment	1.09
**genes UP-regulated after 4 h**			
**#**	**Gene Ontology Term**	**Category**	**Enrichment score**
1	regulation of transcription	Biological process	7.30
2	membrane-enclosed lumen	Cellular compartment	4.93
3	death	Biological process	3.94
4	chordate embryonic development	Biological process	3.88
5	positive regulation of macromolecule metabolic process	Biological process	3.83
**genes DOWN-regulated after 4 h**			
**#**	**Gene Ontology Term**	**Category**	**Enrichment score**
1	ion binding	Molecular function	25.14
2	regulation of transcription	Biological process	19.46
3	regulation of transcription from RNA polymerase II promoter	Biological process	2.77
4	membrane-enclosed lumen	Cellular compartment	2.32
5	chromosome organization	Biological process	1.90
**genes UP-regulated after 24 h**			
**#**	**Gene Ontology Term**	**Category**	**Enrichment score**
1	vesicle-mediated transport	Biological process	2.53
2	enzyme inhibitor activity	Molecular function	2.24
3	cell death	Biological process	2.20
4	nuclease activity	Molecular function	1.97
5	GTPase regulator activity	Molecular function	1.93
**genes DOWN-regulated after 24 h**			
**#**	**Gene Ontology Term**	**Category**	**Enrichment score**
1	extracellular region part/extracellular matrix	Cellular compartment	4.48
2	cellular component morphogenesis	Biological process	2.74
3	carbohydrate binding	Molecular function	2.42
4	nucleotide binding	Molecular function	2.42
5	transmembrane receptor protein tyrosine kinase activity	Molecular function	2.33

Over-represented gene functional categories were identified using the functional annotation clustering tool of DAVID 6.7. Up-regulated and down-regulated genes at each time point were investigated separately. The first 5 gene clusters with their main Gene Ontology term, their category and enrichment score are shown.

**Fig 4:**
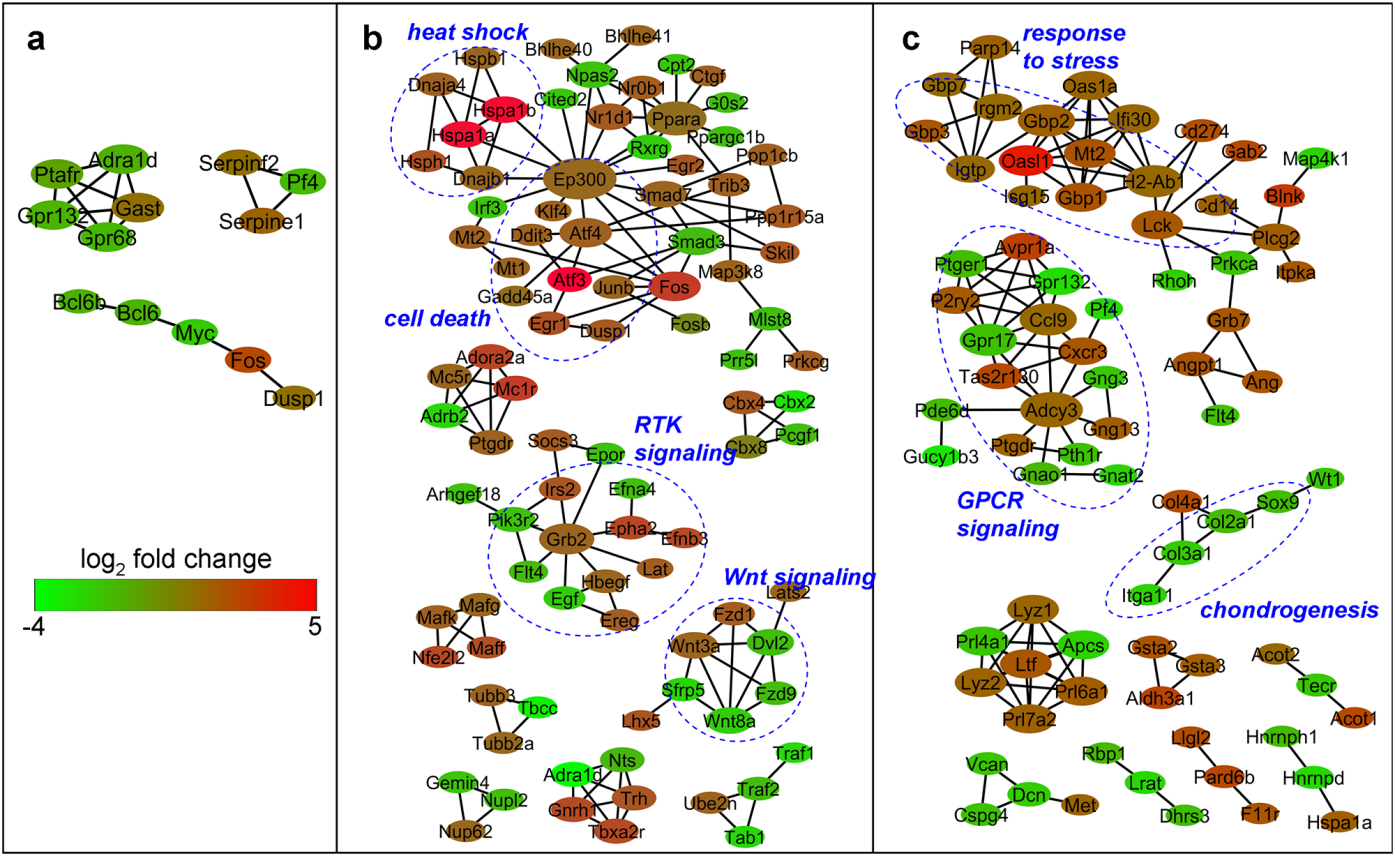
Network of genes modulated by high HP. Network representation of the protein-protein interactions between genes that were significantly (p<0.015) modulated after 1 h (a), 4 h (b) or 24 h (c) of pressurization. Each node represents a protein. Only networks with at least three nodes are shown. The fold change is indicated by the color scale. In order to highlight the more important nodes, the size of the node increases with the number of interacting partners.

Network analysis (Fig 4) revealed several groups of interacting proteins among the significantly modulated genes. After 4 h of pressurization, for instance, groups involved in heat shock, cell death, Wnt signaling and receptor tyrosine kinase (RTK) signaling were found among the networks; after 24 h, groups involved in response to stress, G protein-coupled receptor (GPCR) signaling and chondrogenesis could be observed. Notably, at 4 h, the groups of genes involved in heat shock and cell death were all up-regulated, whereas at 24 h, the group involved in the response to stress was up-regulated while genes implicated in chondrogenesis were down-regulated, indicating overall that, in ATDC5 cells, high pressure first induces a stressful response followed by a reversal of the chondrogenic phenotype.

### Real-time PCR validates most microarray results

**Fig 5:**
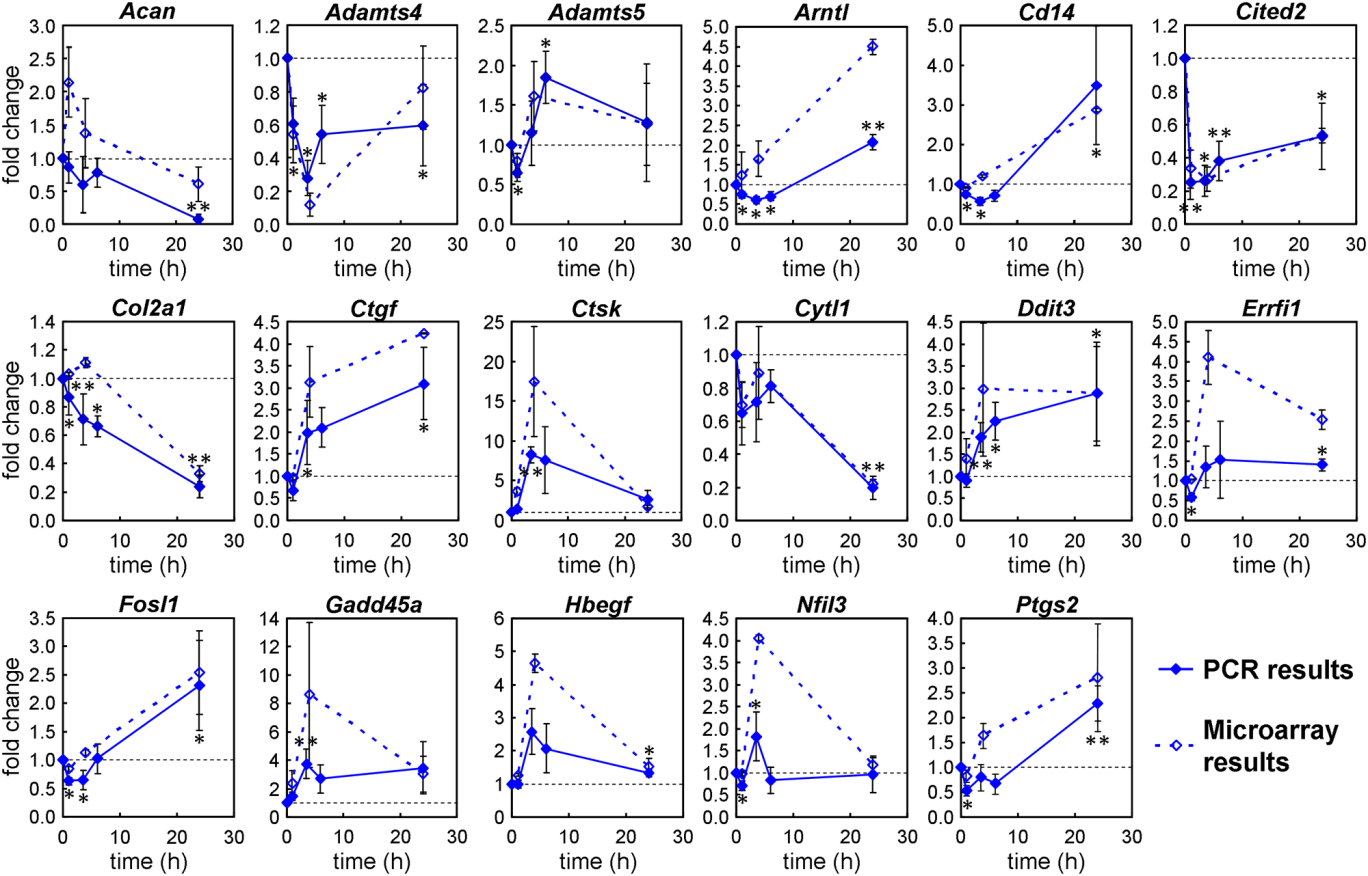
Real-time PCR validation of microarray results. Expression of 17 genes in ATDC5 cells pressurized for 0, 1, 4 or 24 h measured by real-time PCR (solid line) and microarray (dashed line). The plots show the mean +/- SD of three (PCR results) or two (microarray results) independent experiments. *: p< 0.05 and **: p<0.01 compared to unpressurized cells.

To check the validity of the microarray results, 17 genes related to OA and modulated after 1, 4 or 24 h of pressurization were selected and their expression was measured by real-time PCR (Fig 5). Those genes were selected based on the following criteria: 1) microarray fold change > 2; 2) microarray expression level > -5 (to omit weakly expressed genes); 3) potential implication in chondrogenesis or osteoarthritis. Apart from a few genes at certain time points (unlike the microarray results, *Acan* was not up-regulated after 1 h, *Errfi* was not induced after 4 h of HP, *Arntl* and *Cd14* were down-regulated after 4 h of HP), the PCR results closely matched the microarray results. Notably, *Adamts4* was strongly down-regulated and *Adamts5* transiently up-regulated at 4 h; *Acan* and *Col2a1*, which control the synthesis of aggrecan and type 2 collagen, were strongly inhibited after 24 h; *Ctgf* was up-regulated after 4 and up to 24 h; *Ctsk* was strongly induced at 4 h; *Cytl1* was markedly reduced at 24 h; *Ddit3* and *Gadd45a* were up-regulated after 4 and up to 24 h; *Cited2* was down-regulated within 1h and for up to 24 h.

### High HP inhibits ATDC5 cell growth

**Fig 6:**
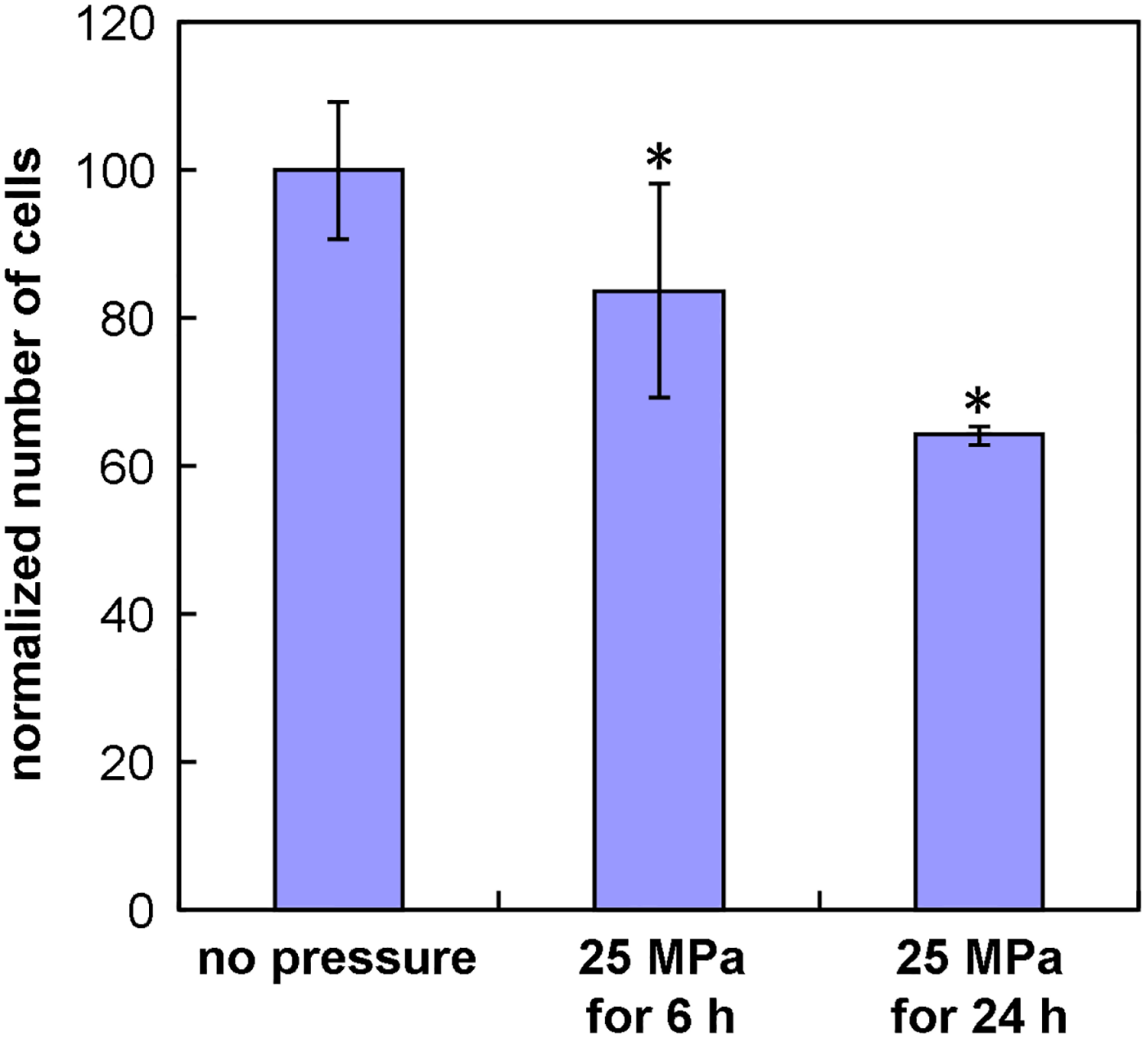
High HP inhibits cell growth. Normalized number of ATDC5 cells after 0, 6 or 24 h under 25 MPa. The graph shows the mean +/- SD of three independent experiments. *: p< 0.05 compared to unpressurized cells.

As genes involved in apoptosis/growth arrest were found to be over-represented among up-regulated genes and the up-regulation of *Ddit3* and *Gadd45a* was confirmed by real-time PCR, we investigated whether high HP affected the growth of ATDC5 cells. A cell count after 0, 6 or 24 h of pressurization showed a significant reduction in the number of cells in pressurized dishes (Fig 6).

### High HP modulates genes similarly to osteoarthritis

Seeing that several genes involved in OA were up-regulated by high pressure, we compared our microarray results with previously published data on surgically induced OA in mice and rats. After comparing genes modulated at least 2-fold in the five selected studies and the present microarray study, we found a total of 449 genes that were modulated in the same direction for at least one time point in both our array and the set of previously reported microarrays, indicating that high HP and OA-inducing surgical procedures affect similar genes (S1 Table). A list of the 30 most modulated genes among those common genes is presented in Table 4.

**Table 4:** Comparison between the present work and previously published studies.

	The present study		Rodent OA model	
Gene symbol	Time point	Fold change	Reference	Fold change
ATF3	4h	37.40	Bateman	4.69
NFE2L2	4h	28.75	Bateman	2.30
RNF183	24h	18.54	Bateman	3.73
CTSK	4h	17.50	Gardiner/Bateman	2.87/3.20
AA467197	4h	14.20	Gardiner	2.03
TRIM65	4h	0.07	Bateman	0.45
HIST1H2AC	24h	13.56	Bateman	2.27
CLEC4D	24h	12.60	Bateman	3.94
ENPP1	24h	12.10	Bateman	3.22
NCMAP	4h	0.09	Gardiner	0.46
COL20A1	24h	0.10	Bateman	0.33
CREB1	4h	10.03	Bateman	2.76
ZFP41	4h	0.10	Bateman	0.39
PPP1R3C	4h	0.10	Gardiner	0.36
FOS	4h	9.33	Bateman	17.51
DNAJB4	4h	9.18	Bateman	2.60
MASP2	24h	0.11	Bateman	0.35
CDHR4	24h	0.11	Bateman	0.38
GADD45A	4h	8.61	Appleton	2.40
SLC30A1	4h	8.57	Bateman	2.01
ARIH1	4h	8.51	Bateman	2.03
ALDH1A3	4h	8.34	Appleton	3.50
ANKZF1	4h	0.12	Bateman	0.41
LYZ2	24h	8.05	Gardiner	2.58
NAV1	4h	0.13	Bateman	0.34
CDADC1	4h	7.99	Bateman	2.15
KLHL15	24h	0.13	Bateman	0.42
FGF11	1h	0.13	Bateman	0.38
D11WSU47E	4h	0.13	Bateman	0.41
ADCK5	4h	0.13	Bateman	0.45

The list shows the 30 most up- or down-regulated genes in our microarray also found modulated in surgically induced OA in mice and rat models. The complete list of 449 genes found in common in our array and the five animal OA studies is given in S1 Table.

## Discussion

The ATDC5 cell line is now a well established cell line to study chondrogenesis and other aspects of cartilage biology [20]. Using a mouse cell line also made it easier to compare our results with previous *in vivo* studies on mouse models of OA, the main model involving destabilization of the medial meniscus (DMM), a procedure that leads to severe osteoarthritis within 8 weeks [21]. In addition, it has been shown that stresses generated in joint cartilage is within one order of magnitude between quadruped species (i.e. mouse or cow) [22]; consequently, it is reasonable to assume that the MPa pressures that are generated in human joints may also be generated in mouse cartilage, despite the different weights.

One limitation to our study was the use of constant pressures, which, at such a high intensity, are unlikely to be found *in vivo*. Indeed, when studying the effects of physiological pressures, cyclical pressures are usually applied; however, high pressures (25 MPa) are just as unlikely to be generated *in vivo* in a cyclical manner. A more physiological approach would have been to apply a single 25 MPa impact pressure to simulate a traumatic injury, which is known to promote the onset of osteoarthritis [23]. However, traumatic injuries *in vivo* trigger a complex response involving several cell types such as immune cells; as our goal was to identify new gene targets of high HP, we decided to use constant pressure and limit the stimulation to 24 h.

Despite the importance of HP in cartilage homeostasis, no whole genome microarray study of the effects of HP has been carried out on chondrocytes or chondrocyte precursors. Sironen et al. used a cDNA array with under 600 probes to identify genes in a human chondrocyte cell line responsive to 30 MPa for 3, 6 or 12 h [12]. Among the genes present in their array, they found 51 modulated genes, while 151 were modulated in our array. Though the overlap was not statistically significant (p = 0.064), 18 genes were found to be modulated in common and in the same direction (representation factor of 1.4), including *Fosl1*, *Hbegf*, *Ddit3* and *Gadd45a*, the latter suggesting that growth arrest/apoptosis may have also been observed in their cells [24].

By looking at the effects of high HP on the whole mouse transcriptome after 1, 4 and 24 h of pressurization, a broad picture of the early response of the cell to high HP could be drawn: an initial stress response followed by arrested growth, increased cell death and a decrease in extra-cellular matrix synthesis, eventually starting the chondrocyte de-differentiation process.

Therefore, among the modulated genes, several genes involved in the physiopathology of OA were identified: *Adamts5*, which was transiently but significantly induced under HP, produces an aggrecanase found responsible for aggrecan degradation in a mouse model of OA [25]. *Arntl* is implicated in chondrocytes’ circadian rhythm, and loss of this gene leads to progressive cartilage degradation in mice [26]. CD14, an antigen usually expressed by immune cells, has also been found in human chondrocytes [27] and may play a role in lymphocyte-mediated cartilage degradation [28]. CITED2 is a transcriptional co-activator known to suppress the expression of the collagen-degrading enzymes MMP-1 and MMP-13 [29][6] and its strong down-regulation further indicates anti-chondrogenic effects of high HP in ATDC5 cells. *Ctgf* was markedly up-regulated after 24 h of pressure and is strongly expressed in osteoarthritic cartilage [30]; although CTGF may be important for cartilage differentiation and maintenance [31], its over-expression has also been shown to cause cartilage damage [32]. *Ctsk*, which produces the collagen-degrading protease cathepsin K, was also transiently but strongly up-regulated by high HP, and knockdown of this enzyme in mice delays the progression of the disease in surgically induced OA [33]. *Cytl1*, strongly down-regulated by 24 h of HP in ATDC5 cells, is a cytokine-like protein involved in chondrogenesis, whose knockdown leads to more severe cartilage destruction in a mouse model of OA [34]. *Ddit3* produces a transcription factor found to participate in cartilage degradation in a mouse model of OA [35]. *Errfi1* deletion in mouse cartilage leads to an OA-like phenotype in the knees, though rarely in other joints [36]. *Fosl1*, markedly up-regulated in our study, produces a FOS-related transcription factor that has been found to be potentially involved in the regulation of gene expression in human osteoarthritic cartilage [37]. *Hbegf*, also transiently up-regulated in our study, produces an EGF-related growth factor, and has been found to be over-expressed in damaged cartilage from patients suffering from OA [38]. *Ptgs2*, which produces cyclooxygenase 2, was also up-regulated after 24 h; its role in OA, however, is still controversial, some studies showing that cyclooxygenase 2 inhibition protects against OA [39] while others show no effect [40].

Surprisingly, *Adamts4*, which is known to be expressed by chondrocytes in osteoarthritic cartilage [41] and also participates in aggrecan degradation in a model of human OA [42], was significantly down-regulated for up to 24 h. However, the respective importance of *Adamts4* and *Adamts5* in the onset and progression of OA is still under investigation. The expression of *Nfil3*, a transcription factor essential to the development of certain immune cells [43], was transiently up-regulated in our study but found down-regulated in osteoarthritic cartilage [44]; the role of NFIL3 in cartilage is still poorly understood and warrants further investigation.

Overall, the microarray results seem to point to osteoarthritic-like effects of high HP on chondrogenic cells. In agreement with the PCR results, several hundred genes modulated by HP were found to be similarly modulated in cartilage from rodent OA models. ATF3, for instance, is a transcription factor, whose expression is increased in OA cartilage and which is involved in the progression of the disease in a mouse OA model [45]. *Nfe2l2* produces a transcription factor, which is known to protect cartilage from degradation in mouse OA models [46]. The overlap between the present results and the previously published microarray studies used for comparison, however, was not statistically significant. This may be due to the different time points used; 24 h of pressurization may only trigger events seen at the very onset of the disease, and longer pressurization periods may be necessary to lead to a more OA-like gene expression profile. Nevertheless, among the newly identified HP-modulated genes, may lie some yet poorly known genes essential to the pathogenesis of OA, which further studies will help to uncover.

In this study, no growth factors or hormones were added to the medium to promote ATDC5 differentiation into mature chondrocytes before HP was applied. However, ATDC5 cells were seeded at a relatively high density and were fully confluent for one day before the start of the pressurization. Real-time PCR confirmed that they thus clearly expressed *Sox9*, *Acan* and *Col2a1*. Moreover, we found that 25 MPa strongly inhibits *Acan* and *Col2a1* expression; this is similar to the effects of high HP (20 to 50 MPa) reviewed by Elder et al. [4], which include a decrease in collagen and aggrecan gene expression in bovine chondrocytes. In addition, one technical difficulty with using differentiated ATDC5 cells is that the cell population obtained after differentiation may not be homogeneous, making the results difficult to interpret. Though investigating the effects of pressure on primary (differentiated) chondrocytes may be an interesting future study, we believe the present study using a homogeneous population of ATDC5 cells yielded meaningful data on the mechanobiology of hydrostatic pressure.

As cells do not contain any gas phase and are considered almost incompressible, physiological HP does not induce any visible deformation of cell compartments or the cell as a whole, making the mechanotransduction mechanisms difficult to identify. However, within 1 h, several hundreds of genes responded to pressurization, and it is quite possible that several independent mechanisms could be implicated in the sensing of HP: for instance, HP may affect the conformation of a pressure-sensitive protein, though studies showing visible changes in protein conformation usually deal with pressures above 100 MPa [47]; HP may alter membrane fluidity and indirectly affect the conformation or the binding of signaling molecules [48]; HP could also alter forces within the membrane by increasing its bending rigidity [49], generating forces sufficient to trigger a mechano-chemical response.

As physiological pressures are known to be beneficial to cartilage, and high HP induces changes in cells reminiscent of those observed in OA, identifying the pressure mechanotransducer(s) in chondrocytes and their downstream pathways may be a key to better understand and prevent the progression of OA.

## Supporting information

S1 Table Genes modulated similarly in the present study and in rodent OA models. List of all the 449 genes found to be modulated in both the present study and in the five studies by Gardiner et al., Bateman et al., Loeser et al., Appleton et al. and Wei et al. on cartilage samples from surgically induced OA in mice and rats. The table shows the gene symbol, the strongest fold change in our data and the time point, as well as the fold change and study, in which the gene was also modulated.
(DOCX)
