# Supplementary material for: High hydrostatic pressure induces pro-osteoarthritic changes in cartilage precursor cells: A transcriptome analysis
Source: PLoS One. 2017 Aug 16;12(8):e0183226. doi: 10.1371/journal.pone.0183226 (PMC5558982; doi:10.1371/journal.pone.0183226)
Supplement: S1 Table — List of all the 449 genes found to be modulated in both the present study and in the five studies by Gardiner et al., Bateman et al., Loeser et al., Appleton et al. and Wei et al. on cartilage samples from surgically induced OA in mice and rats. The table shows the gene symbol, the strongest fold change in our data and the time point, as well as the fold change and study, in which the gene was also modulated. (DOCX) [file pone.0183226.s001.docx]

**S1 Table: genes modulated similarly in the present study and in rodent OA models.** List of all the 449 genes found to be modulated in both the present study and in the five studies by Gardiner et al., Bateman et al., Loeser et al., Appleton et al. and Wei et al. on cartilage samples from surgically induced OA in mice and rats. The table shows the gene symbol, the strongest fold change in our data and the time point, as well as the fold change and study, in which the gene was also modulated.

|  | **The present study** | | **Rodent OA model** | |
| --- | --- | --- | --- | --- |
| **Gene symbol** | **Time point** | **Fold change** | **Reference** | **Fold change** |
| ATF3 | 4h | 37.40 | Bateman | 4.69 |
| NFE2L2 | 4h | 28.75 | Bateman | 2.30 |
| RNF183 | 24h | 18.54 | Bateman | 3.73 |
| CTSK | 4h | 17.50 | Gardiner/Bateman | 2.87/3.20 |
| AA467197 | 4h | 14.20 | Gardiner | 2.03 |
| TRIM65 | 4h | 0.07 | Bateman | 0.45 |
| HIST1H2AC | 24h | 13.56 | Bateman | 2.27 |
| CLEC4D | 24h | 12.60 | Bateman | 3.94 |
| ENPP1 | 24h | 12.10 | Bateman | 3.22 |
| NCMAP | 4h | 0.09 | Gardiner | 0.46 |
| COL20A1 | 24h | 0.10 | Bateman | 0.33 |
| CREB1 | 4h | 10.03 | Bateman | 2.76 |
| ZFP41 | 4h | 0.10 | Bateman | 0.39 |
| PPP1R3C | 4h | 0.10 | Gardiner | 0.36 |
| FOS | 4h | 9.33 | Bateman | 17.51 |
| DNAJB4 | 4h | 9.18 | Bateman | 2.60 |
| MASP2 | 24h | 0.11 | Bateman | 0.35 |
| CDHR4 | 24h | 0.11 | Bateman | 0.38 |
| GADD45A | 4h | 8.61 | Appleton | 2.40 |
| SLC30A1 | 4h | 8.57 | Bateman | 2.01 |
| ARIH1 | 4h | 8.51 | Bateman | 2.03 |
| ALDH1A3 | 4h | 8.34 | Appleton | 3.50 |
| ANKZF1 | 4h | 0.12 | Bateman | 0.41 |
| LYZ2 | 24h | 8.05 | Gardiner | 2.58 |
| NAV1 | 4h | 0.13 | Bateman | 0.34 |
| CDADC1 | 4h | 7.99 | Bateman | 2.15 |
| KLHL15 | 24h | 0.13 | Bateman | 0.42 |
| FGF11 | 1h | 0.13 | Bateman | 0.38 |
| D11WSU47E | 4h | 0.13 | Bateman | 0.41 |
| ADCK5 | 4h | 0.13 | Bateman | 0.45 |
| TLE2 | 24h | 0.13 | Bateman | 0.41 |
| SERPINE1 | 1h | 7.41 | Wei | 2.80 |
| FBN1 | 24h | 0.14 | Bateman | 0.45 |
| SPEER3 | 24h | 7.15 | Bateman | 2.08 |
| PYCR1 | 24h | 0.14 | Bateman | 0.40 |
| EGF | 4h | 0.14 | Appleton | 0.50 |
| CPM | 1h | 0.14 | Gardiner | 0.40 |
| PLP1 | 24h | 0.14 | Bateman | 0.46 |
| GPRASP2 | 24h | 0.14 | Bateman | 0.47 |
| ATF1 | 4h | 6.93 | Bateman | 2.25 |
| SPTY2D1 | 4h | 6.74 | Bateman | 2.46 |
| CCNI | 4h | 6.69 | Bateman | 3.67 |
| LIMK2 | 24h | 0.15 | Bateman | 0.47 |
| TCF25 | 24h | 0.15 | Bateman | 0.45 |
| CMTM7 | 4h | 0.16 | Bateman | 0.49 |
| S100A5 | 4h | 6.22 | Bateman | 4.06 |
| TAB1 | 4h | 0.16 | Bateman | 0.33 |
| VPS4B | 4h | 6.20 | Bateman | 2.11 |
| SP4 | 4h | 6.16 | Bateman | 2.30 |
| CTSH | 24h | 6.12 | Bateman | 5.21 |
| RAB3A | 4h | 0.16 | Bateman | 0.30 |
| TLK2 | 4h | 6.08 | Bateman | 2.18 |
| ADAMTSL2 | 24h | 0.17 | Bateman | 0.15 |
| SLC1A2 | 24h | 5.82 | Wei | 0.00 |
| LGALS4 | 24h | 0.17 | Bateman | 0.25 |
| CNNM3 | 1h | 0.17 | Bateman | 0.36 |
| SOX5 | 24h | 0.17 | Bateman | 0.43 |
| STEAP2 | 24h | 5.61 | Bateman | 2.12 |
| PRKAG2 | 4h | 0.18 | Bateman | 0.35 |
| CYS1 | 4h | 0.18 | Bateman | 0.14 |
| MTMR7 | 24h | 5.47 | Bateman | 4.06 |
| FGF18 | 4h | 5.46 | Wei | 3.50 |
| C1QTNF2 | 4h | 5.34 | Gardiner | 2.04 |
| MATN2 | 24h | 5.29 | Gardiner | 2.17 |
| TNIK | 24h | 0.19 | Bateman | 0.40 |
| OBSL1 | 24h | 0.19 | Bateman | 0.33 |
| LRCH4 | 4h | 0.19 | Bateman | 0.47 |
| SMPD3 | 4h | 0.19 | Bateman | 0.32 |
| KRTCAP3 | 24h | 0.20 | Bateman | 0.35 |
| FOXP1 | 24h | 0.20 | Bateman | 0.43 |
| ZFP78 | 4h | 0.20 | Bateman | 0.49 |
| RAMP2 | 24h | 4.97 | Bateman | 2.66 |
| MZF1 | 4h | 0.20 | Bateman | 0.41 |
| RNFT2 | 24h | 0.20 | Bateman | 0.47 |
| SMARCA1 | 24h | 0.20 | Bateman | 0.37 |
| SEL1L | 24h | 4.91 | Bateman | 2.89 |
| TM9SF1 | 4h | 0.20 | Bateman | 0.48 |
| HSPH1 | 4h | 4.82 | Bateman | 3.33 |
| ZMAT1 | 4h | 0.21 | Bateman | 0.32 |
| APBA1 | 24h | 0.21 | Wei | 0.48 |
| ZFP207 | 4h | 4.79 | Bateman | 2.08 |
| OCIAD2 | 24h | 4.78 | Gardiner | 2.46 |
| UCMA | 24h | 0.21 | Gardiner | 0.37 |
| ABCG1 | 24h | 4.73 | Bateman | 6.42 |
| CSRNP1 | 4h | 4.71 | Bateman | 3.98 |
| NMB | 4h | 4.70 | Bateman | 2.05 |
| IFRD1 | 4h | 4.68 | Bateman | 2.15 |
| CHD6 | 24h | 0.21 | Bateman | 0.29 |
| HBEGF | 4h | 4.64 | Bateman | 8.29 |
| TMCC3 | 24h | 4.63 | Bateman | 24.64 |
| PARD3B | 4h | 0.22 | Bateman | 0.50 |
| COQ10B | 4h | 4.57 | Bateman | 3.63 |
| CSPG4 | 24h | 0.22 | Bateman | 0.34 |
| KCTD11 | 4h | 0.22 | Bateman | 0.46 |
| PRR5L | 4h | 0.22 | Bateman | 0.28 |
| MMRN2 | 4h | 4.52 | Bateman | 6.22 |
| ANXA6 | 4h | 0.22 | Bateman | 0.35 |
| SLC25A27 | 4h | 0.22 | Bateman | 0.50 |
| ARNTL | 24h | 4.50 | Bateman | 4.11 |
| CYTL1 | 24h | 0.22 | Gardiner/Loeser | 0.34/0.46 |
| YOD1 | 4h | 4.50 | Bateman | 2.78 |
| CITED2 | 4h | 0.22 | Appleton | 0.40 |
| PDE4A | 4h | 0.22 | Bateman | 0.36 |
| TMEM140 | 4h | 0.23 | Bateman | 0.38 |
| DUSP6 | 4h | 4.38 | Bateman | 3.40 |
| SGMS2 | 4h | 4.29 | Bateman | 3.85 |
| CHN2 | 24h | 4.29 | Bateman | 5.19 |
| SDF4 | 4h | 4.28 | Bateman | 2.05 |
| SEMA3A | 4h | 0.24 | Appleton | 0.50 |
| GRIK5 | 24h | 0.24 | Bateman | 0.36 |
| RNF4 | 4h | 4.20 | Bateman | 2.16 |
| NCAM1 | 24h | 0.24 | Bateman | 0.24 |
| PVRL3 | 4h | 4.19 | Bateman | 2.23 |
| MAP1LC3B | 1h | 4.18 | Bateman | 2.19 |
| FAM89A | 24h | 4.17 | Bateman | 6.37 |
| MACROD1 | 24h | 0.24 | Bateman | 0.50 |
| SFI1 | 4h | 0.24 | Bateman | 0.39 |
| F11R | 24h | 4.16 | Bateman | 4.43 |
| PSAT1 | 4h | 4.16 | Appleton | 2.70 |
| ERRFI1 | 4h | 4.10 | Bateman | 6.00 |
| GLI2 | 4h | 0.24 | Bateman | 0.24 |
| SKIL | 4h | 4.07 | Bateman | 4.01 |
| FAM84B | 24h | 4.06 | Bateman | 3.31 |
| NFIL3 | 4h | 4.05 | Bateman | 5.37 |
| FCGR3 | 1h | 4.05 | Appleton | 3.00 |
| JMJD1C | 4h | 0.25 | Bateman | 0.39 |
| MPND | 24h | 0.25 | Bateman | 0.40 |
| RFXANK | 24h | 0.25 | Bateman | 0.44 |
| CFD | 1h | 0.25 | Gardiner/Bateman | 0.33/0.16 |
| KRR1 | 4h | 3.99 | Bateman | 2.14 |
| GHR | 4h | 0.25 | Bateman | 0.20 |
| SPINK2 | 24h | 3.95 | Gardiner | 2.01 |
| GCNT2 | 4h | 3.95 | Bateman | 2.97 |
| LRFN3 | 4h | 0.25 | Bateman | 0.45 |
| SH2D5 | 4h | 3.93 | Bateman | 2.51 |
| LRRN4CL | 4h | 3.92 | Gardiner | 2.35 |
| CDK17 | 4h | 3.92 | Bateman | 2.17 |
| USP40 | 1h | 0.26 | Bateman | 0.34 |
| MFSD6 | 24h | 3.90 | Bateman | 3.37 |
| PPP4R2 | 4h | 3.89 | Bateman | 2.04 |
| DHRS3 | 24h | 0.26 | Bateman | 0.42 |
| SULT1E1 | 4h | 3.82 | Bateman | 3.28 |
| PDLIM3 | 24h | 0.26 | Gardiner | 0.42 |
| PLXNA3 | 24h | 0.26 | Bateman | 0.40 |
| RBMS3 | 24h | 0.26 | Bateman | 0.29 |
| MET | 24h | 3.80 | Bateman | 3.74 |
| ZGPAT | 4h | 0.26 | Bateman | 0.45 |
| FEM1C | 4h | 3.76 | Bateman | 3.34 |
| PTP4A1 | 4h | 3.76 | Bateman | 3.01 |
| HAGHL | 4h | 0.27 | Bateman | 0.46 |
| CADM1 | 24h | 3.69 | Bateman | 5.12 |
| IQGAP1 | 4h | 3.67 | Bateman | 3.13 |
| AFF3 | 24h | 0.27 | Bateman | 0.40 |
| NLGN2 | 1h | 0.27 | Bateman | 0.34 |
| LIMCH1 | 24h | 0.27 | Bateman | 0.38 |
| DNAJA4 | 4h | 3.64 | Bateman | 2.41 |
| PPP1R15A | 4h | 3.64 | Bateman | 2.07 |
| C8G | 24h | 0.28 | Bateman | 0.38 |
| FBXO10 | 4h | 0.28 | Bateman | 0.44 |
| CSTAD | 4h | 0.28 | Bateman | 0.33 |
| TTLL3 | 24h | 0.28 | Bateman | 0.32 |
| SOCS3 | 4h | 3.59 | Bateman | 2.82 |
| SYNJ2BP | 4h | 3.59 | Bateman | 2.05 |
| HSPA4 | 4h | 3.56 | Bateman | 2.96 |
| RUNX1 | 4h | 3.56 | Bateman | 2.62 |
| BC022687 | 24h | 3.56 | Bateman | 2.58 |
| CHD9 | 24h | 3.55 | Bateman | 2.57 |
| RAB1 | 1h | 3.54 | Bateman | 4.25 |
| VAT1L | 24h | 3.54 | Gardiner | 2.31 |
| SFN | 4h | 3.52 | Bateman | 3.21 |
| ZRANB3 | 24h | 0.28 | Bateman | 0.42 |
| FAM188B | 4h | 0.28 | Bateman | 0.47 |
| MCL1 | 4h | 3.52 | Bateman | 4.40 |
| WDR61 | 4h | 3.52 | Bateman | 2.58 |
| FAM160A2 | 1h | 0.28 | Bateman | 0.46 |
| PPT1 | 24h | 3.51 | Bateman | 2.66 |
| RPS20 | 24h | 3.51 | Bateman | 3.48 |
| TPM2 | 24h | 0.28 | Gardiner | 0.29 |
| ZHX3 | 4h | 0.29 | Bateman | 0.50 |
| FAM180A | 24h | 3.49 | Gardiner | 2.42 |
| CENPP | 24h | 0.29 | Gardiner | 0.41 |
| SBNO2 | 24h | 3.42 | Bateman | 2.45 |
| CCDC159 | 4h | 0.29 | Bateman | 0.48 |
| AHDC1 | 4h | 0.29 | Bateman | 0.48 |
| OGN | 24h | 0.30 | Bateman | 0.25 |
| ATF4 | 4h | 3.36 | Bateman | 2.44 |
| GTF2E1 | 1h | 0.30 | Bateman | 0.44 |
| CCDC37 | 24h | 0.30 | Bateman | 0.34 |
| PDE4D | 24h | 3.34 | Bateman | 2.29 |
| PHKA2 | 1h | 0.30 | Bateman | 0.26 |
| FZD1 | 4h | 3.33 | Appleton | 2.00 |
| MYLPF | 24h | 0.30 | Gardiner | 0.19 |
| AVL9 | 4h | 3.31 | Bateman | 2.23 |
| RBBP7 | 4h | 3.30 | Bateman | 2.08 |
| PUS7L | 1h | 0.30 | Bateman | 0.46 |
| PLEKHA1 | 24h | 3.30 | Bateman | 2.21 |
| HEATR5A | 4h | 3.30 | Bateman | 2.13 |
| TEKT2 | 24h | 0.30 | Bateman | 0.34 |
| FAM161A | 4h | 0.30 | Bateman | 0.26 |
| ZFP13 | 4h | 0.31 | Bateman | 0.49 |
| LAMP2 | 1h | 3.26 | Bateman | 2.00 |
| FZD9 | 4h | 0.31 | Bateman | 0.32 |
| IER3 | 4h | 3.22 | Bateman | 5.97 |
| GM4876 | 24h | 0.31 | Bateman | 0.42 |
| TICAM1 | 4h | 0.31 | Bateman | 0.48 |
| CDO1 | 24h | 0.31 | Gardiner | 0.40 |
| SAT2 | 24h | 0.31 | Bateman | 0.35 |
| EDA2R | 4h | 3.19 | Bateman | 2.05 |
| CCDC69 | 24h | 3.18 | Bateman | 2.68 |
| PCGF1 | 4h | 0.31 | Gardiner | 0.47 |
| CPEB4 | 4h | 3.17 | Bateman | 2.06 |
| LATS2 | 4h | 3.17 | Bateman | 2.05 |
| CCDC77 | 4h | 0.32 | Bateman | 0.49 |
| GPR135 | 4h | 0.32 | Bateman | 0.45 |
| IQCE | 1h | 0.32 | Bateman | 0.40 |
| PLSCR1 | 24h | 3.16 | Bateman | 2.43 |
| RGMA | 4h | 0.32 | Bateman | 0.38 |
| TWSG1 | 24h | 3.14 | Bateman | 3.74 |
| OSBP2 | 1h | 0.32 | Bateman | 0.46 |
| OLFML3 | 4h | 3.13 | Gardiner | 2.27 |
| GBP2 | 24h | 3.13 | Bateman | 5.48 |
| ARHGEF18 | 4h | 0.32 | Bateman | 0.30 |
| COL2A1 | 24h | 0.32 | Bateman | 0.42 |
| FEZ2 | 24h | 3.11 | Bateman | 2.41 |
| DGKQ | 4h | 0.32 | Bateman | 0.44 |
| SIL1 | 24h | 0.32 | Bateman | 0.42 |
| IL1RL1 | 24h | 3.08 | Bateman | 3.65 |
| AGPAT9 | 4h | 3.07 | Bateman | 3.97 |
| BCORL1 | 4h | 0.33 | Bateman | 0.49 |
| ADAMTS5 | 4h | 3.07 | Bateman | 2.28 |
| SLC35A3 | 4h | 3.07 | Bateman | 2.11 |
| SLC43A1 | 4h | 0.33 | Bateman | 0.37 |
| SNED1 | 4h | 0.33 | Bateman | 0.39 |
| N4BP3 | 4h | 0.33 | Bateman | 0.36 |
| ARHGEF9 | 24h | 0.33 | Appleton | 0.50 |
| TOP2A | 1h | 3.03 | Appleton | 2.00 |
| CD14 | 24h | 3.00 | Bateman | 4.71 |
| PRRX1 | 1h | 0.33 | Bateman | 0.21 |
| KLHL21 | 4h | 3.00 | Bateman | 4.37 |
| STK17B | 1h | 2.99 | Appleton | 2.40 |
| RND3 | 4h | 2.99 | Bateman | 3.40 |
| HYI | 24h | 0.34 | Bateman | 0.36 |
| SMPDL3A | 24h | 2.96 | Bateman | 2.91 |
| LIN7C | 4h | 2.96 | Bateman | 2.88 |
| ZFAND2A | 24h | 2.96 | Bateman | 2.59 |
| LDLR | 1h | 2.93 | Wei | 3.30 |
| SLC9A8 | 4h | 0.34 | Bateman | 0.47 |
| DDX3X | 4h | 2.93 | Bateman | 2.25 |
| KCTD9 | 4h | 2.92 | Bateman | 2.05 |
| SCD1 | 24h | 2.91 | Bateman | 4.61 |
| EPC1 | 4h | 2.90 | Bateman | 2.40 |
| SCAI | 24h | 0.35 | Bateman | 0.45 |
| MED25 | 24h | 0.35 | Bateman | 0.40 |
| KLF4 | 4h | 2.89 | Appleton | 2.20 |
| LYSMD3 | 4h | 2.88 | Bateman | 2.60 |
| CCT4 | 4h | 2.88 | Bateman | 2.09 |
| PKN2 | 4h | 2.88 | Bateman | 2.09 |
| RAB5A | 4h | 2.87 | Bateman | 2.25 |
| EAF2 | 24h | 2.87 | Bateman | 2.22 |
| LIN54 | 4h | 2.86 | Bateman | 2.01 |
| ST3GAL1 | 24h | 2.85 | Bateman | 2.28 |
| MTHFSD | 4h | 0.35 | Bateman | 0.33 |
| BMPER | 4h | 0.35 | Bateman | 0.38 |
| PLCXD2 | 24h | 2.85 | Bateman | 2.29 |
| DEFB11 | 1h | 2.84 | Bateman | 5.97 |
| SRR | 24h | 2.84 | Bateman | 2.76 |
| PTPN22 | 24h | 2.84 | Bateman | 7.92 |
| C4B | 24h | 0.35 | Bateman | 0.33 |
| NRIP3 | 24h | 2.82 | Bateman | 2.13 |
| INHBB | 24h | 2.81 | Wei | 2.60 |
| COL5A3 | 4h | 0.36 | Bateman | 0.29 |
| GPM6B | 24h | 2.81 | Gardiner | 3.52 |
| PTGS2 | 24h | 2.81 | Gardiner/Bateman | 2.64/32.81 |
| PRDX1 | 1h | 2.80 | Bateman | 2.89 |
| ANKRD26 | 4h | 0.36 | Bateman | 0.41 |
| HNRNPR | 4h | 2.79 | Bateman | 3.01 |
| JMJD8 | 24h | 0.36 | Bateman | 0.37 |
| ANKRD12 | 4h | 2.77 | Bateman | 2.04 |
| SH2B2 | 4h | 0.36 | Bateman | 0.50 |
| KLF9 | 4h | 2.77 | Bateman | 2.67 |
| OSBPL7 | 4h | 0.36 | Bateman | 0.39 |
| HECTD2 | 24h | 0.36 | Gardiner | 0.48 |
| MTF1 | 24h | 2.75 | Bateman | 3.25 |
| FBXL18 | 4h | 0.37 | Bateman | 0.42 |
| APC | 4h | 2.73 | Bateman | 2.95 |
| TMBIM4 | 24h | 2.73 | Bateman | 3.15 |
| RANBP2 | 4h | 2.72 | Bateman | 2.53 |
| SLBP | 1h | 2.72 | Bateman | 2.19 |
| BCL7A | 4h | 0.37 | Gardiner | 0.35 |
| CUL1 | 4h | 2.71 | Bateman | 2.37 |
| PFKFB4 | 4h | 0.37 | Bateman | 0.36 |
| PPIC | 24h | 0.37 | Bateman | 0.35 |
| REST | 4h | 2.70 | Bateman | 2.11 |
| RNF19B | 4h | 2.70 | Bateman | 2.62 |
| TRIM7 | 4h | 0.37 | Bateman | 0.44 |
| PYGM | 1h | 0.37 | Bateman | 0.33 |
| CACNA2D3 | 24h | 0.37 | Wei | 0.26 |
| OLFML2B | 24h | 2.65 | Gardiner | 2.82 |
| ANGPTL2 | 24h | 2.65 | Gardiner | 2.05 |
| CCDC127 | 4h | 2.64 | Bateman | 2.28 |
| BHLHE40 | 4h | 2.64 | Bateman | 3.03 |
| BCL10 | 4h | 2.64 | Bateman | 2.71 |
| SARDH | 24h | 0.38 | Bateman | 0.31 |
| NFIX | 24h | 0.38 | Bateman | 0.49 |
| BTG1 | 4h | 2.63 | Bateman | 5.83 |
| CFLAR | 24h | 2.63 | Bateman | 2.80 |
| DOCK6 | 1h | 0.38 | Bateman | 0.31 |
| PAFAH1B3 | 24h | 0.38 | Appleton | 0.50 |
| CRK | 4h | 2.63 | Bateman | 2.21 |
| ZFP286 | 4h | 0.38 | Bateman | 0.37 |
| ECHDC3 | 4h | 0.38 | Bateman | 0.40 |
| MAP3K12 | 4h | 0.38 | Bateman | 0.38 |
| ACTA1 | 24h | 0.38 | Gardiner | 0.26 |
| CAR3 | 1h | 0.38 | Gardiner | 0.35 |
| MAP2K2 | 24h | 0.38 | Bateman | 0.50 |
| SEMA3E | 24h | 2.60 | Bateman | 8.47 |
| ANGPT2 | 4h | 2.60 | Bateman | 4.68 |
| SOCS2 | 24h | 2.59 | Gardiner | 2.28 |
| GREM1 | 4h | 0.39 | Gardiner | 0.17 |
| IER5 | 4h | 2.58 | Appleton | 3.20 |
| DNAJA1 | 4h | 2.58 | Bateman | 2.73 |
| DNAJB1 | 4h | 2.58 | Bateman | 2.04 |
| TMED5 | 4h | 2.56 | Bateman | 2.86 |
| KCND2 | 1h | 0.39 | Wei | 0.44 |
| SATB1 | 4h | 0.39 | Bateman | 0.46 |
| B3GALT4 | 4h | 0.39 | Bateman | 0.37 |
| PDZK1IP1 | 1h | 0.39 | Bateman | 0.46 |
| PIEZO2 | 1h | 2.55 | Gardiner | 3.40 |
| MSI2 | 24h | 0.39 | Bateman | 0.50 |
| STOML1 | 4h | 0.39 | Bateman | 0.42 |
| AFG3L1 | 24h | 0.39 | Bateman | 0.36 |
| FOSL1 | 24h | 2.54 | Bateman | 5.43 |
| GNA13 | 4h | 2.54 | Bateman | 3.60 |
| PRKG1 | 24h | 0.39 | Gardiner | 0.47 |
| ZYG11B | 1h | 0.40 | Bateman | 0.48 |
| SH3BGRL2 | 4h | 2.53 | Bateman | 5.44 |
| ITGB1 | 4h | 2.53 | Bateman | 2.51 |
| IL17RD | 24h | 2.52 | Gardiner/Bateman | 2.13/4.21 |
| HP | 4h | 2.52 | Gardiner | 2.23 |
| ACTR5 | 4h | 0.40 | Bateman | 0.42 |
| APPBP2 | 4h | 2.51 | Wei | 2.30 |
| SPATA2L | 4h | 0.40 | Bateman | 0.40 |
| PCNX | 4h | 2.50 | Bateman | 2.40 |
| PIK3CD | 1h | 0.40 | Bateman | 0.21 |
| ACTC1 | 24h | 0.40 | Gardiner | 0.18 |
| RASSF2 | 4h | 0.40 | Bateman | 0.34 |
| TOM1L2 | 1h | 0.40 | Bateman | 0.45 |
| PHF3 | 1h | 0.40 | Bateman | 0.41 |
| SCARA3 | 24h | 0.40 | Bateman | 0.29 |
| ZFP414 | 24h | 0.40 | Bateman | 0.42 |
| ZFP612 | 4h | 0.40 | Bateman | 0.37 |
| GIGYF2 | 4h | 2.47 | Bateman | 3.88 |
| ARGLU1 | 24h | 0.41 | Bateman | 0.42 |
| VCAM1 | 24h | 2.46 | Gardiner | 2.25 |
| NCK2 | 1h | 2.46 | Bateman | 2.38 |
| RYBP | 4h | 2.46 | Bateman | 3.56 |
| KITL | 4h | 2.46 | Appleton | 5.30 |
| APOC4 | 1h | 0.41 | Wei | 0.30 |
| MBD1 | 4h | 0.41 | Bateman | 0.45 |
| CBX8 | 1h | 0.41 | Bateman | 2.06 |
| PPP1R15B | 4h | 2.42 | Bateman | 2.17 |
| NRAP | 1h | 0.41 | Gardiner | 0.43 |
| 2010111I01RIK | 24h | 2.41 | Loeser | 2.50 |
| DCP1B | 4h | 0.42 | Bateman | 0.47 |
| LDB3 | 4h | 0.42 | Gardiner | 0.39 |
| NOP56 | 1h | 0.42 | Bateman | 0.33 |
| COL5A1 | 4h | 2.39 | Loeser | 2.62 |
| NT5E | 24h | 2.39 | Bateman | 16.00 |
| RCN3 | 24h | 0.42 | Bateman | 0.40 |
| TGFB1I1 | 4h | 0.42 | Bateman | 0.48 |
| STARD4 | 24h | 0.42 | Bateman | 0.45 |
| E2F2 | 4h | 2.38 | Gardiner | 2.08 |
| LRP4 | 24h | 2.38 | Appleton | 2.30 |
| TNFAIP6 | 4h | 2.38 | Wei | 2.30 |
| FGFR2 | 24h | 0.42 | Appleton | 0.40 |
| IVD | 24h | 0.42 | Bateman | 0.46 |
| B3GNT2 | 4h | 2.37 | Bateman | 2.70 |
| NEDD4L | 24h | 2.37 | Bateman | 2.16 |
| RABGGTB | 4h | 2.37 | Bateman | 4.21 |
| RTN4 | 4h | 2.36 | Bateman | 3.23 |
| KLHL17 | 4h | 0.42 | Bateman | 0.47 |
| OSR2 | 24h | 2.36 | Gardiner | 2.50 |
| PTPRO | 1h | 2.36 | Bateman | 3.78 |
| SOCS6 | 4h | 2.35 | Bateman | 2.28 |
| KLHL31 | 4h | 0.43 | Gardiner | 0.40 |
| B4GALT1 | 4h | 2.33 | Bateman | 3.32 |
| CHDH | 1h | 0.43 | Gardiner | 0.45 |
| LRMP | 4h | 0.43 | Bateman | 0.50 |
| CLTB | 24h | 2.32 | Bateman | 2.86 |
| CRIM1 | 24h | 2.32 | Bateman | 2.73 |
| XIRP2 | 24h | 0.43 | Gardiner | 0.35 |
| MEX3C | 4h | 2.31 | Bateman | 3.05 |
| SKAP2 | 24h | 2.30 | Bateman | 2.58 |
| FHIT | 1h | 0.43 | Wei | 0.43 |
| SLC5A3 | 24h | 0.43 | Appleton | 0.40 |
| CMBL | 24h | 2.28 | Bateman | 3.17 |
| FOXJ3 | 4h | 2.28 | Gardiner | 2.95 |
| MICALL2 | 4h | 0.44 | Bateman | 0.27 |
| ENGASE | 4h | 0.44 | Bateman | 0.44 |
| FAM109A | 4h | 0.44 | Bateman | 0.46 |
| RIIAD1 | 24h | 0.44 | Bateman | 0.48 |
| RIOK1 | 4h | 0.44 | Bateman | 0.49 |
| KCNK5 | 4h | 2.26 | Bateman | 4.30 |
| CDC73 | 4h | 2.25 | Bateman | 2.15 |
| CSPP1 | 4h | 0.44 | Bateman | 0.48 |
| DFNA5 | 24h | 2.25 | Bateman | 3.11 |
| SYPL | 4h | 2.25 | Bateman | 2.88 |
| RNF170 | 1h | 0.45 | Bateman | 0.43 |
| ATP6V0B | 4h | 2.25 | Bateman | 2.09 |
| MOCS1 | 4h | 0.45 | Bateman | 0.43 |
| XYLT2 | 4h | 0.45 | Bateman | 0.34 |
| RBBP4 | 1h | 2.23 | Bateman | 2.12 |
| DLX5 | 24h | 0.45 | Appleton | 0.40 |
| SGIP1 | 4h | 0.45 | Bateman | 0.33 |
| SUV420H1 | 4h | 2.22 | Gardiner | 2.16 |
| CROCC | 4h | 0.45 | Bateman | 0.36 |
| PPAP2B | 24h | 2.22 | Bateman | 2.89 |
| CALM1 | 4h | 2.22 | Bateman | 2.40 |
| MAP3K2 | 1h | 2.21 | Bateman | 2.31 |
| TMEM64 | 4h | 2.21 | Bateman | 3.75 |
| ATP6AP2 | 24h | 2.20 | Bateman | 6.96 |
| FBP1 | 24h | 0.45 | Appleton | 0.50 |
| CEBPD | 24h | 2.19 | Wei | 2.50 |
| RASGEF1A | 1h | 0.46 | Bateman | 0.21 |
| METTL6 | 4h | 2.16 | Bateman | 2.02 |
| ABCD4 | 24h | 0.46 | Bateman | 0.36 |
| TCEAL3 | 1h | 0.46 | Bateman | 0.39 |
| ING4 | 4h | 0.46 | Bateman | 0.48 |
| ARL8B | 4h | 2.16 | Bateman | 2.18 |
| FTO | 24h | 0.46 | Bateman | 0.38 |
| PFKP | 4h | 2.15 | Bateman | 3.43 |
| FAM188A | 4h | 2.15 | Bateman | 2.09 |
| ATF6 | 24h | 2.14 | Bateman | 2.03 |
| EXD1 | 4h | 0.47 | Bateman | 0.46 |
| FGGY | 24h | 0.47 | Bateman | 0.46 |
| SLC31A1 | 4h | 2.13 | Bateman | 2.38 |
| SGPL1 | 24h | 2.12 | Bateman | 2.16 |
| ARAF | 24h | 0.47 | Bateman | 0.47 |
| PPM1J | 24h | 2.12 | Bateman | 2.88 |
| GALNT10 | 24h | 2.11 | Gardiner | 2.07 |
| TGOLN1 | 24h | 2.11 | Bateman | 2.57 |
| KIF5A | 1h | 0.48 | Bateman | 0.28 |
| FAM179B | 4h | 2.09 | Bateman | 2.14 |
| TRIB1 | 1h | 2.09 | Bateman | 2.71 |
| PRPF40A | 4h | 2.09 | Bateman | 2.54 |
| NACAD | 24h | 0.48 | Bateman | 0.47 |
| TTC28 | 24h | 0.49 | Bateman | 0.31 |
| ENAH | 24h | 0.49 | Wei | 0.26 |
| RSRC1 | 24h | 0.49 | Bateman | 0.33 |
